# Study the characterization and thermoelectrical properties of the polyvinyl alcohol / Polyaniline polymer blend thick films

**DOI:** 10.1186/s11671-025-04267-x

**Published:** 2025-10-29

**Authors:** M. A. Morad, M. S. Abo Ghazala, M. G. El-Shaarawy, M. E. Gouda, T. Y. Elrasasi

**Affiliations:** 1 Physics Department, Faculty of Science, Menoufia University, Shebin El-Koom, Egypt; 2 Physics Department, Faculty of Science, Benha University, Benha, Egypt

**Keywords:** PVA, Polyaniline, Thermoelectric materials

## Abstract

This study investigated the impact of the introduction of Polyaniline (PANi) on the structural, thermal stability, electrical, and thermoelectric properties of polyvinyl alcohol (PVA)_1−x_ Polyaniline (PANi)_x_ blend composites, (where x = 0, 10, 20, and 30 wt.%). The prepared blends were synthesized via the casting technique. The process of polymerizing polyaniline (PANi) was executed in a methodical and ordered manner. The obtained films from these blends are analyzed to assess their surface characteristics and structural morphology through elemental analysis, scanning electron microscopy (SEM), and Fourier-transform infrared spectroscopy (FTIR), as well as their thermal properties via thermogravimetric analysis (TGA) and differential scanning calorimetry (DSC). The PVA hydrogen bonding facilitates the uniform dispersion of PANi among the chains of PVA, which increases the amorphous structure of the prepared films. The surface of the pure PVA film is characterized by a smooth surface. However, a mixture of nanofibers with slightly white porous spongy morphology patches appeared in the PANi-doped films. Furthermore, the incorporation of PANi into the PVA matrix improves the thermal stability of the prepared films. The impact of PANi on the electrical properties, Seebeck Coefficient, and thermal conductivity of the prepared composites is evaluated using the four-probe direct technique and laser flash measurements. SEM images reveal a heterogeneous distribution of conductive PANi particles within the continuous PVA matrix. A notable aspect of this investigation is the significant increment in the DC electrical conductivity of the blend films at room temperature, which increases from 2.08 × 10^^−12^ S/m for the pure PVA film to 0.08 S/m for the film containing 30 wt.% PANi. The Seebeck Coefficient decreases with loading the PANi due to the increase in the charge carrier concentration. Concurrently, there was a slight enhancement in thermal conductivity, increasing from 0.1304 W m^−1^ K^−1^ to 0.362 W m⁻^1^ K^−1^ for the 0 wt.% and 30 wt.% films, respectively. The findings suggest a polymer blend with significant potential for thermoelectrical applications, exhibiting high electrical and low thermal conductivity, which is advantageous for thermoelectric applications.

## Introduction

The search for a renewable energy source is a global challenge because of rising energy costs and global warming associated with fossil fuel sources. For this purpose, thermoelectric (TE) energy converters are increasingly interesting because these solid-state devices can transform heat from sources into electric power. Consequently, there has been an increasing interest from governments and research institutions in high-performance thermoelectric (TE) materials, which can directly and reversibly transform heat into electrical energy, offering promising solutions for waste heat recovery, refrigeration, and power generation [1].

The thermoelectric phenomena are fundamentally associated with three key phenomena: the Seebeck effect, the Peltier effect, and the Thomson effect, which were discovered by three scientists independently [2, 3]. Historically, the search for novel thermoelectric materials has primarily emphasized inorganic semiconductor materials [3]. However, the interest in more flexible, lightweight, cheaper, feasible, operatable in a wide temperature range, and environmentally friendly materials has drawn scientists’ attention more to organic materials [4]. However, the efficiency of thermoelectric devices is not high enough to rival the Carnot efficiency [5]. The evaluation of the TE property is conducted through a dimensionless Figure of Merit (ZT) [6]:

1 $$\varvec{ZT}~ = \sigma \varvec{S}^{2} {\raise0.7ex\hbox{$\varvec{T}$} \!\mathord{\left/ {\vphantom {\varvec{T} \varvec{k}}}\right.\kern-\nulldelimiterspace} \!\lower0.7ex\hbox{$\varvec{k}$}}$$

where σ is the electrical conductivity, *k* is the thermal conductivity, T is the absolute temperature, and S is the Seebeck coefficient [6, 7]:

2 $$\varvec{S } = \varvec{ }\Delta {\varvec{V}}/\Delta {\varvec{T}}$$

where ∆V is the potential difference, and ∆T is the temperature difference.

The main principle of thermoelectric materials involves the optimization of various competing properties to achieve a high-performance thermoelectric material with a high ZT (Figure of Merit) value. Maximize the thermoelectric figure of merit (ZT), requires several conflicting characteristics such as a high electrical conductivity like that of crystalline metals, low thermal conductivity similar to that of glass, and a high Seebeck coefficient comparable to that of crystalline semiconductors. It is challenging to find a material that has these conflicting properties. Insulators and semiconductors exhibit high Seebeck coefficients and low thermal conductivities; however, they are characterized by low carrier concentrations and diminished electrical conductivity. In contrast, metals are identified by high electrical and thermal conductivities with low Seebeck coefficient, however, the Seebeck coefficient in metals and certain semiconductor materials is primarily attributed to the presence of free charge carriers, which are responsible for the transport of both charge and thermal energy [8–10].

By applying a temperature gradient to the thermoelectric material, the charge carriers diffuse from the hot to the cold end building up an accumulated charge (negative for electrons, e– at the hot end, and positive for holes, h + at the cold end), producing an electrostatic potential (voltage). This phenomenon is known as the Seebeck effect, which is the basis of thermoelectric generators. The maximum thermoelectric efficiency of a thermoelectric generator is given by [5, 10]:

3 $$\varvec{\varphi }_{{{\mathbf{max}}}} = \varvec{ }\frac{{\varvec{ P}_{{{\mathbf{out}}}} \varvec{ }}}{{{\varvec{Q}}_{{{\mathbf{in}}}} }}$$

*where Q*_in_ is the amount of heat entering the material, and *P*_out_ is the generated electrical power by the material, including the heat losses.

Historically, metals and their alloys, like Ni, Cu, Bi, Al, and Sb, along with semiconductors such as PbTe and Bi_2_Te_3_ were the main TE materials choice of the scientists. Although these materials can be very efficient in thermoelectric applications, they are often expensive to produce, heavy, and not widely available. Many studies have been conducted to find alternative TE materials, focusing on replacing metal and alloy-based thermoelectric materials with semiconductors and polymers. Polymer TE materials have gained a lot of interest lately because their physical and chemical properties can be easily adjusted through simple changes at the molecular level. As a result, researchers have looked into organic polymers, their doped composites, and polymer blends as potential thermoelectric materials. Such as; Polyacetylene [11], Polypyrroles [Polyanilines [12], Polythiophenes [13], Poly(2,7-carbazoles) [14], Polyvinyl alcohol (PVA), Polyaniline (PANi) [15]], Polythiophene(PTH) [16], Poly(3,4 ethylene dioxythiophene): Poly(styrene sulfonate)/tosylate (PEDOT: PSS, PEDOT Tos) [17], Polyacetylene (PA), Polypyrrole (PPY), Polycarbazoles (PC), Polyphenylenevinylene (PPV), and their derivatives [18, 19].

The polymer blend is a type of material analogous to metal alloys, where two or more polymers are combined to create a new substance that displays unique physical properties. These blends can be categorized into three primary types: immiscible polymer blends, and miscible polymer blends [20, 21]. The exploration of conductive blended polymer materials has garnered significant attention due to their capacity to yield materials with advantageous characteristics such as their alternating double and single bonds that include π electrons in the backbone chain which made a strong demand for using them as insulation in films or sheets to create a conductive layer for electroplating semiconductors. They can be applied in electronic devices, printed circuit boards, solar cells, photovoltaic systems, batteries, and thermoelectricity [22]. Linking of functional groups in the conductive polymers played a significant role in their thermoelectrical materials, such as the creation of conduction paths, controlling the charge carrier scattering, and phonons movement making them the attention of the researchers on the thermoelectricity and numerous applications [23–25].

Among the various organic materials, conducting polymers are particularly notable for their adjustable electrical characteristics, minimal thermal conductivity, and straightforward synthesis process [26]. While their electrical conductivities can rival those of metals, conducting polymers offer several advantages, including lightweight properties, corrosion resistance, flexibility, and cost-effectiveness [15, 27, 28]. These materials are increasingly utilized in a variety of applications, such as solar cells, batteries, actuators, sensors, electromagnetic shielding, thermoelectricity, and microelectronic devices. In a comprehensive analysis, researchers focused their efforts on the study of conductive polymer blends [15, 27, 29, 30].

Conducting polymers such as Polyaniline (PANi) has recently become an area of widespread interest in organic electronics due to their potential application in energy conversion systems such as thermoelectricity and solar cells [30–32]. However, significant challenges associated with the effective use of polyaniline (PANi) include its inadequate mechanical properties and limited solubility in both aqueous and organic solvents [33]. Enhancements in the characteristics of polyaniline can be realized through the development of composites and nanocomposites involving aniline, or by blending it with commercially available polymers or inorganic materials that provide improved mechanical and optical properties, as well as enhanced stability and processability of PANi [20, 34–37].

Polyvinyl alcohol (PVA) is a flexible, water-soluble, and mechanically robust polymer with excellent film-forming abilities and chemical resistance. PVA’s insulating nature and mechanical properties make it an ideal candidate for blending with PANI to create composite materials with enhanced thermoelectric properties. PVA is an insulating polymer with a low thermal conductivity of 0.13 Wm^–1^ K^–1^ [38–40]. Due to these properties, PVA polymer composites have attracted attention for different applications.

Ayat Abd-Elsalam et al. [41], have reported on the synthesis and thermoelectric properties of pristine polyaniline and polyaniline/carbon nanotubes network nanocomposite. They prepared the PANi via a facile chemical oxidative polymerization process to aniline, in which 30 wt% multiwall carbon nanotubes were incorporated in situ. For their thermoelectric performance, the fabricated composite exhibited a clear increase in the Seebeck coefficient and power factor to reach 141 µV/K and 0.004 µW/mK^2^, respectively at 95 °C.

Reza Amirabad et al. [42], also investigated the electrical and thermoelectrical properties of the fabrics coated with polyaniline/carbon nanotube. Flexible polyester/yarn thermoelectric fabrics were coated with polyaniline/carbon nanotube (PANi/CNT) nanocomposite, were fabricated by sequential processing: (1) polyaniline/carbon nanotube nanocomposites preparation by a one-step polymerization and (2) dip coating of a mixture solution of CNT-doped PANI on a polyester/yarn fabric. Nanocomposites were synthesized with various CNT contents (0.5, 2.5, 5, and 10 wt. %). They reported a significant improvement in both electrical conductivity and the Seebeck coefficient with the CNT introduction. The electrical conductivity increased from 0.011 to 0.1345 S/cm for the CNT concentration 0.5 to 10 wt%. The highest Seebeck coefficient of 11.4 μV/K was observed for the sample containing 5 wt.% CNT at 338 K, while the fabric coated with nanocomposite containing 10 wt% CNT obtained the maximum power factor of about 1.598 × 10^–3^ µW/mK^2^.

Blending PVA with PANi presents a novel approach to improving PANi’s flexibility, processability, and mechanical strength while maintaining or enhancing its thermoelectric performance. Such blends can offer a fine-tuning between electrical conductivity and thermal insulation, both critical for achieving a high thermoelectric figure of merit (ZT) [26, 43]. Additionally, the hydrogen bonding interactions between PVA and PANi can lead to better dispersion of the conducting polymer in the matrix, improving the overall performance of the composite. In recent years, there has been growing interest in developing PVA/PANi blends as potential thermoelectric materials [4, 26, 44]. These blends can be optimized by adjusting the PANi concentration, the degree of doping in PANi, and the structural morphology of the composite. Furthermore, incorporating nanofillers or using hybrid structures may offer further improvements in thermoelectric performance by enhancing electrical conductivity while reducing thermal conductivity.

In this study, we focus on synthesizing and characterizing the PVA/PANi blend for thermoelectric applications. By exploring different compositions and fabrication methods, we aim to investigate how the structural, thermal, and electrical properties of these blends impact their thermoelectric performance. The ultimate goal is to evaluate the feasibility of PVA/PANi composites as flexible, lightweight thermoelectric materials for use in low-temperature ranges.

## Experimental

### Materials

PVA(Polyvinyl alcohol) [(C_2_H_4_O)_n_ ( M_w_ 89,000–98,000, 99 + % hydrolyzed), CAS-No. 9002–89-5, and EC-No. 618–340-9] was purchased from Sigma-Aldrich Chemie GmbH in the United States. Aniline Monomer C_6_H_5_NH_2_ (93.129 g/mol) was purchased from QualiChem’s Laboratories in India, while Hydrochloric acid (HCL) (35% AR) and Ammonium peroxidisulfate ((NH_4_)_2_S_2_O_8_) (98% AR) were purchased from Merck Darmstadt Germany and LOBA CHEMIE PVT. LTD from India, respectively.

### Preparation of polyaniline

Using the chemical oxidative polymerization method, Polyaniline (PANi) powder was synthesized through polymerization of aniline monomer in the presence of hydrochloric acid HCL (acts as a catalyst) and using ammonium peroxide-sulfate ((NH_4_)_2_S_2_O_8_) (acts as an oxidizing agent) in a 4:1 monomer/oxidant molar ratio [17]. In a 250 mL volumetric flask at a temperature of ≈ –10 °C, 20 mL of aniline was dissolved in 40 mL of 1 mol L^–1^ aqueous solution of hydrochloric acid (3.3 ml conc HCl + 36.7 ml H_2_O). In a separate flask,12 g of ammonium peroxide-sulfate ((NH_4_)_2_S_2_O_8_) was dissolved in 160 mL of 1 mol L^–1^ hydrochloric acid aqueous solution. For 2 h and under constant stirring, the acid solution of ((NH_4_)_2_S_2_O_8_) was dripped into aniline acid solution carefully and slowly. In this process, the reaction medium was kept at ≈ –10 °C left at rest to polymerize for 24 h. The reaction medium changed color, going through tones of brown, blue, and green, and a solid deposit was formed at the bottom of the reaction flask. The green sediment was filtered, washed three times using 100 ml portions of 1 mol L^–1^ HCl solution and similarly with acetone and dried at room temperature for 72 h. Under these conditions, the polymer obtained is in a doped state (PANi-HCl) (emeraldine salt) slightly soluble in water.

### Synthesis of (PVA_1-x_/PANi_x_) blend

**Fig. 1:**
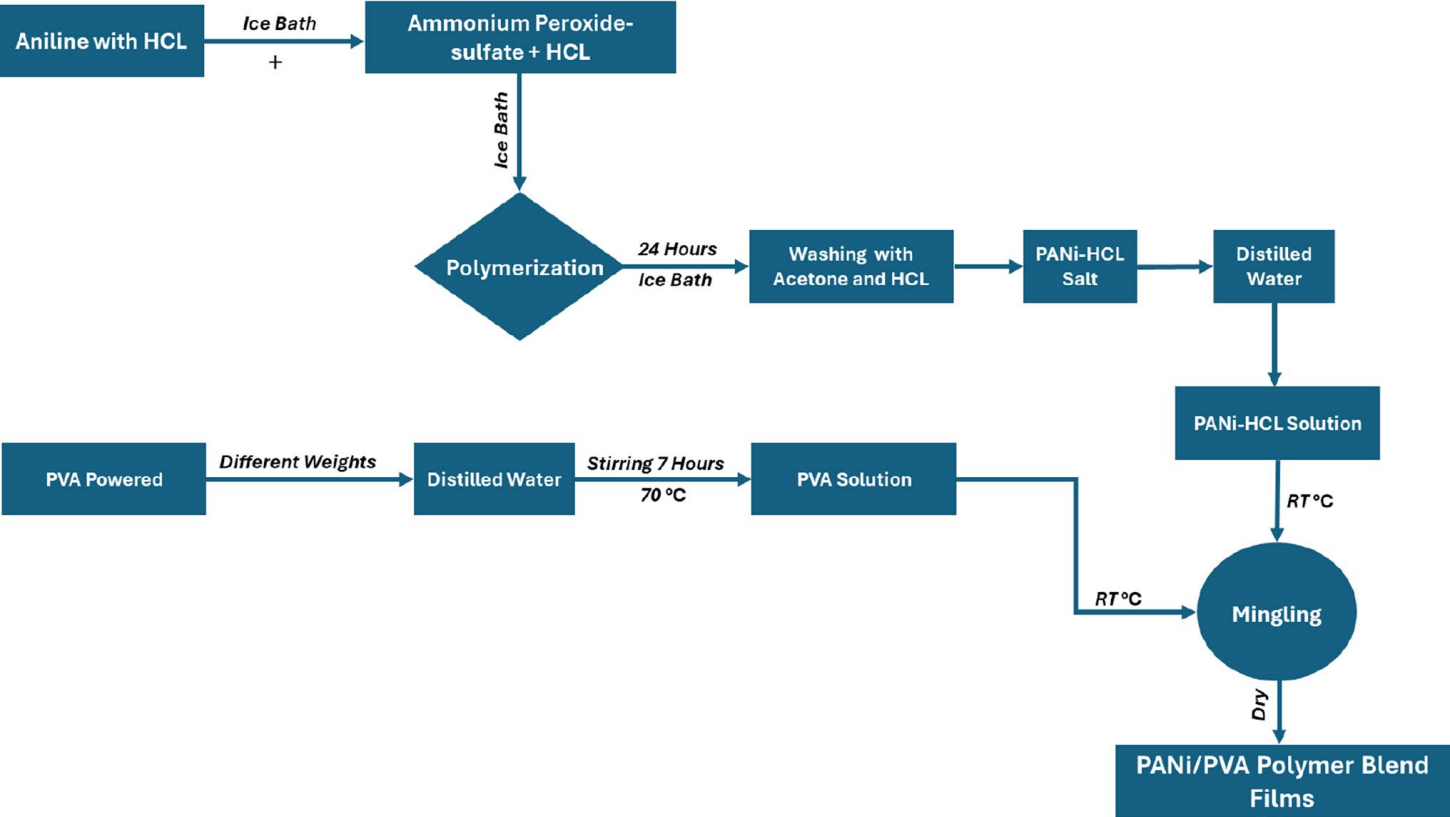
The preparation of the PVA_1-x_/PANi_x_ polymer blend films where x = 0, 10, 20, 30 wt.%

Different weights of the prepared PANi-HCL (0.1 g, 0.2 g, and 0.3 g) were dissolved in 20 mL distilled water for 6 h using stirring at room temperature, followed by 1 h at room temperature using an ultrasonic probe. Separately, at 70 °C under persistent stirring for 8 h, 0.9 g, 0.8 g, and 0.7 g of PVA were dissolved in 20 mL distilled water, then left the PVA solutions for 4 h to reach room temperature. PVA_1-x_/PANi_x_ polymer blends were prepared via the casting method after PANi solutions mingled with the PVA solutions (at room temperature) under continual stirring for 12 h, then cast the films in Petri dishes and left for 3 days. Figure 1 shows the preparation of the polymer blend films. Table 1 shows the polymer blend film elements.

**Table 1:** Elements weight in grams for the prepared films

PANi wt.%	PVA content in the blend (gm)	PANi content in the blend (gm)	Film name
0	1	0	0 wt.%
10	0.9	0.1	10 wt.%
20	0.8	0.2	20 wt.%
30	0.7	0.3	30 wt.%

### Characterization techniques

Fourier-transform infrared Spectra (FT-IR) scans were recorded in an FT-IR Bruker ALPHA II infrared spectrometer in the wave number range (400–4000 cm^−1^) to the prepared polymer blend films to the molecular interactions.

The structural properties of prepared films were studied using an XRD Rigaku Miniflex 600 X-Ray Diffractometer. The diffraction system is based on a Cu tube anode with a voltage ≈ of 40 kV, current ≈ 30 mA, and wavelength Ka1 = 1.5418 Å has been used to calculate the crystallographic spacing utilizing the Bragg’s law (λ = 2*d*sinθ).

The surface morphology of the films was examined by analyzing the scanning electron microscopy (SEM) images and the energy dispersive spectroscopy analysis (EDX) was recorded using a scanning electron microscope (JEOL released the JCM-7000 Benchtop SEM), operated at 20 kV.

Setaram Themys carried out thermogravimetric analyses of the prepared films at a heating rate of 10 °C /min under a nitrogen atmosphere from room temperature to 600 °C.

The thermal conductivity of the prepared films was measured by Linseis LZT Laser Flash Meter – (Model LFA 1000) in a temperature range of 20 °C to 120 °C with a heating rate of 1 °C/min out-of-plan the films. The films’ out-of-plane DC electrical conductivity and Seebeck Coefficient were measured within a temperature range of 20 °C to 120 °C using the Linseis LSR-3 system with a heating rate of 1 °C /min. Films of the pristine composites with a thickness ≈ of 0.2 mm, length ≈ of 30 mm, and width of ≈ 1.5 mm were prepared for the electrical and thermal conductivity measurements.

## Results and discussion

### X-ray diffraction

**Fig. 2:**
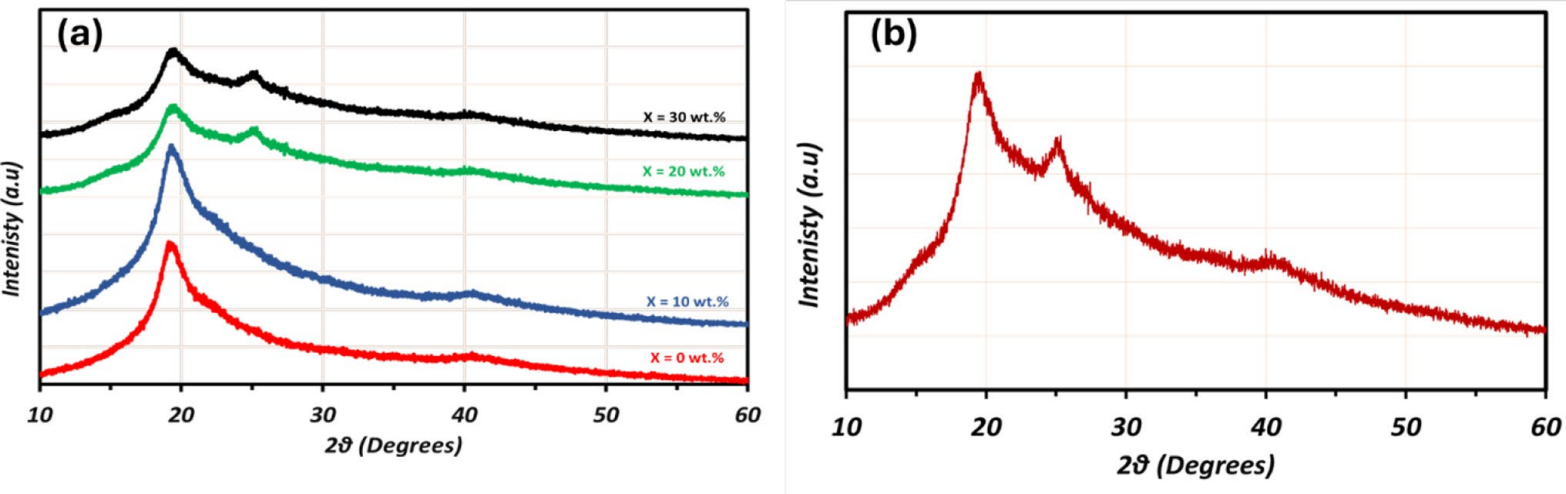
The XRD pattern of; **a** the prepared PVA_1-x_/PANi_x_ polymer blend films where x = 0, 10, 20, 30 wt.%, and **b** Pure PANi salt

Figure 2 shows the X-ray diffraction pattern of the synthesized (PVA_1-x_PANi_x_) polymer blend and the pure PANi at room temperature in the scanning range 10° ≤ 2θ ≤ 60°. The pure PANi pattern shows the two peaks centered at 2θ = 20° and 2θ = 25° indicating the partial amorphous structure of PANi as shown in Fig. 1b [45]. In Fig. 2a, The PVA patterns exhibit a prominent hump of 2θ = 19.7° at corresponds to a mixture of (101) and (10ĺ) reflection planes with 4.6 nm d-spacing corresponding to the phase hexagonal crystallinity phase nature of the pure PVA matrix due to the strong intermolecular hydrogen bonding between PVA chains [31].

In the doped PVA/PANi films, an additional broad peak was observed at 2θ = 25° clarifying the amorphous nature of the PANi [37, 46]. The prepared film 10 wt. % PANi, the pattern shows the same structure as the pure PVA film, which demonstrates the amorphous behaviour of the PANi in the present film. This broad peak is ascribed to the periodicity perpendicular to the PANi chain. It is characteristic of Van der Waals distances between stacks of polyaniline rings. The increase in PANi concentration, the more broadness and decreased intensity in peaks are observed in the patterns [47]. Notably, the peak intensity decreases with the introduction of the PANi into the PVA matrix reflecting the influence of the amorphous structure of the PANi salt in the films.

### FT-IR spectra

**Fig. 3:**
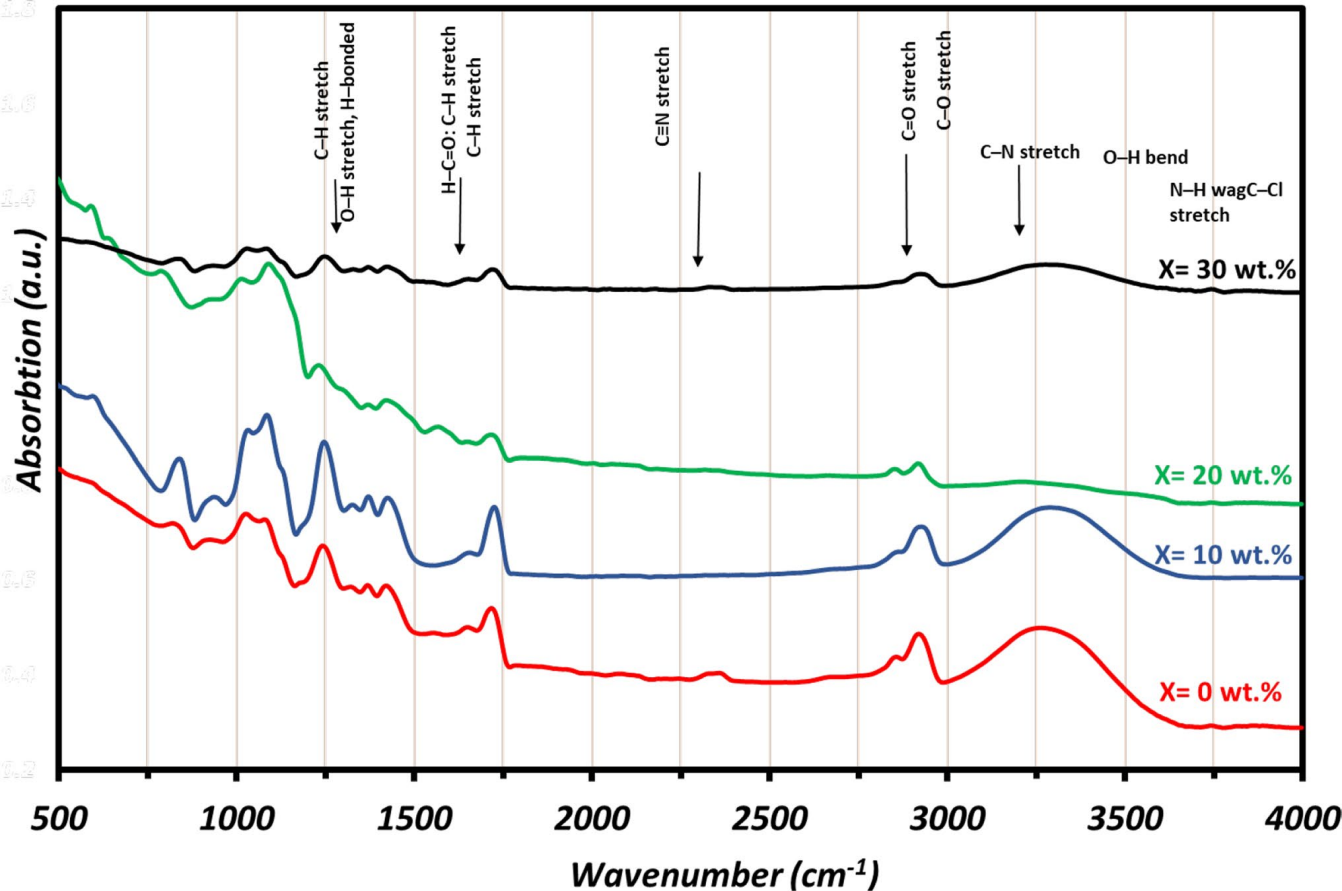
The FTIR spectra patterns of the prepared films

Fourier transform infrared spectroscopy (FT-IR**)** was used to investigate the functional groups and identify the interactions in the prepared films as shown in Fig. 3. In the pure PVA spectrum, it can be observed that the peaks situated at 3264, 2920, 1710, and 1648 cm^−1^ correspond to stretching vibrations of –OH groups, C–H, C = O, and C = C, respectively. Additional exhibited bands at 1417 and 1079 cm^−1^ which assigned to the stretching vibrations of –OH and C = O, respectively [47, 48]. The peaks at 910 and 836 cm^−1^ also refer to C–C stretching and CH_2_ stretching, respectively. Finally, the peak at 1317 cm^−1^ is due to the coupling of the O–H vibration 1417 cm^−1^ with the C–H wagging vibrations [48]. In the doped PVA/PANi films spectrum, new bands appear at 1240, and 1157 cm^−1^ belong to the stretching and bending of C–N and C–H bonds of PANi [49]. The intensity of these beaks increases with increasing the PANi concentration in the films. The N–H stretching band at 3400–3500 cm^−1^ of PANi overlaps with the O–H bond of PVA stretching and, therefore cannot be separately assigned. Moreover, after incorporating PANi within the PVA matrix, most peak positions weaken the bond strength in PVA and PANi due to electron withdrawing, which might arise from the strong electrostatic interactions between PANi and PVA [50]. Besides, the intensity of the PVA peaks was reduced after introducing the PANi, suggesting the breaking of the hydrogen bonding of PVA and the spread of PANi among the chains of PVA which increased the amorphous structure of the prepared samples and the uniform dispersion of the PANi in the PVA matrix [51, 52].

### SEM morphology and energy-dispersive X-ray (EDX)

**Fig. 4:**
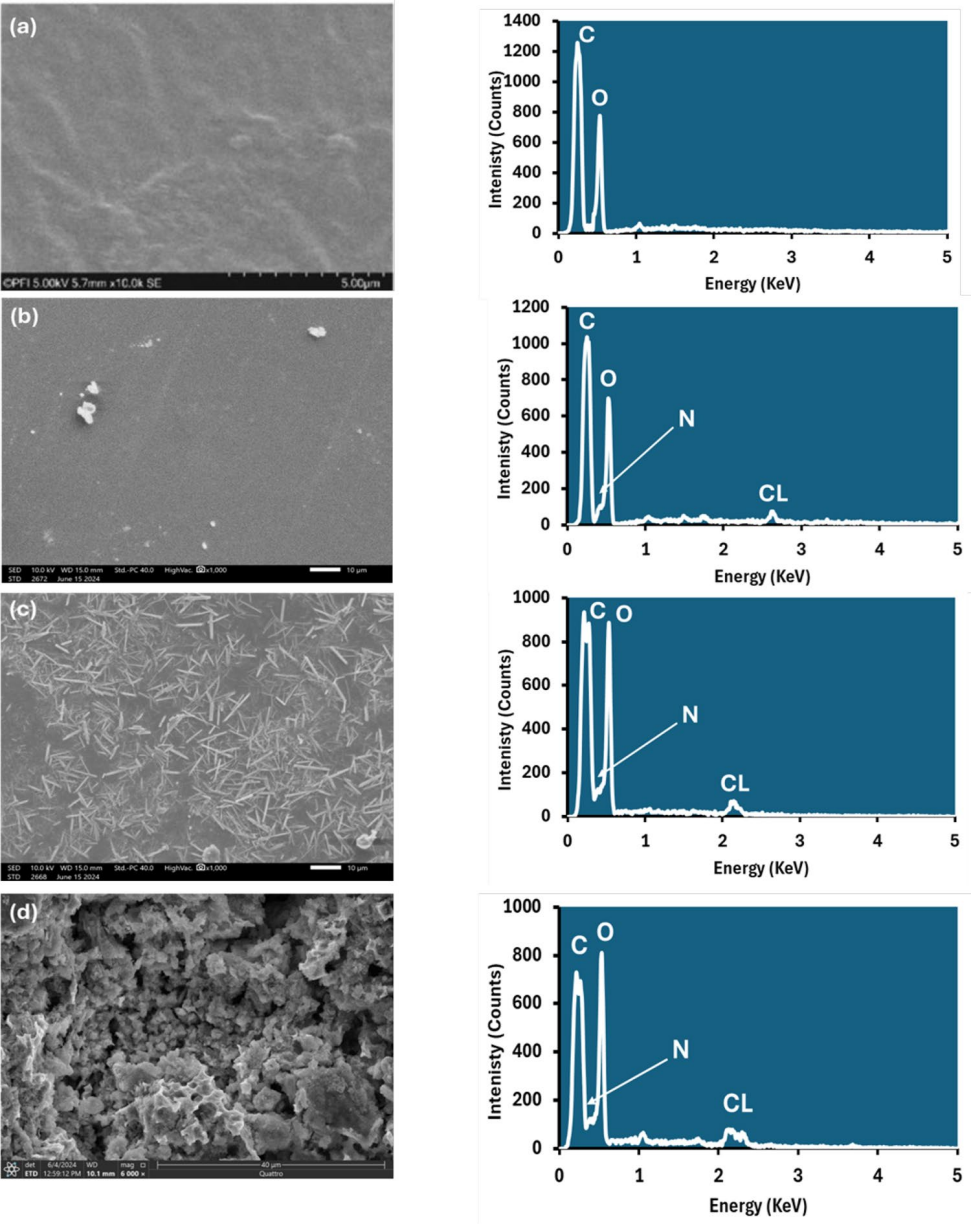
SEM images and EDX analysis of (PVA_1-x_PANi_x_) prepared films (where **a** x = 0, **b** x = 10, **c** x = 20, and **d** x = 30 wt. %)

Scanning electron microscope (SEM) has been used to study the compatibility between the PVA and PANi through the detection of phase separations, interfaces, and surface morphology which has a reflection on the other physical parameters. Figure 4 shows the surface SEM photographs of all investigated samples. The pure PVA matrix is characterized by a smooth surface and does not exhibit any preferential orientation [52].

**Fig. 5:**
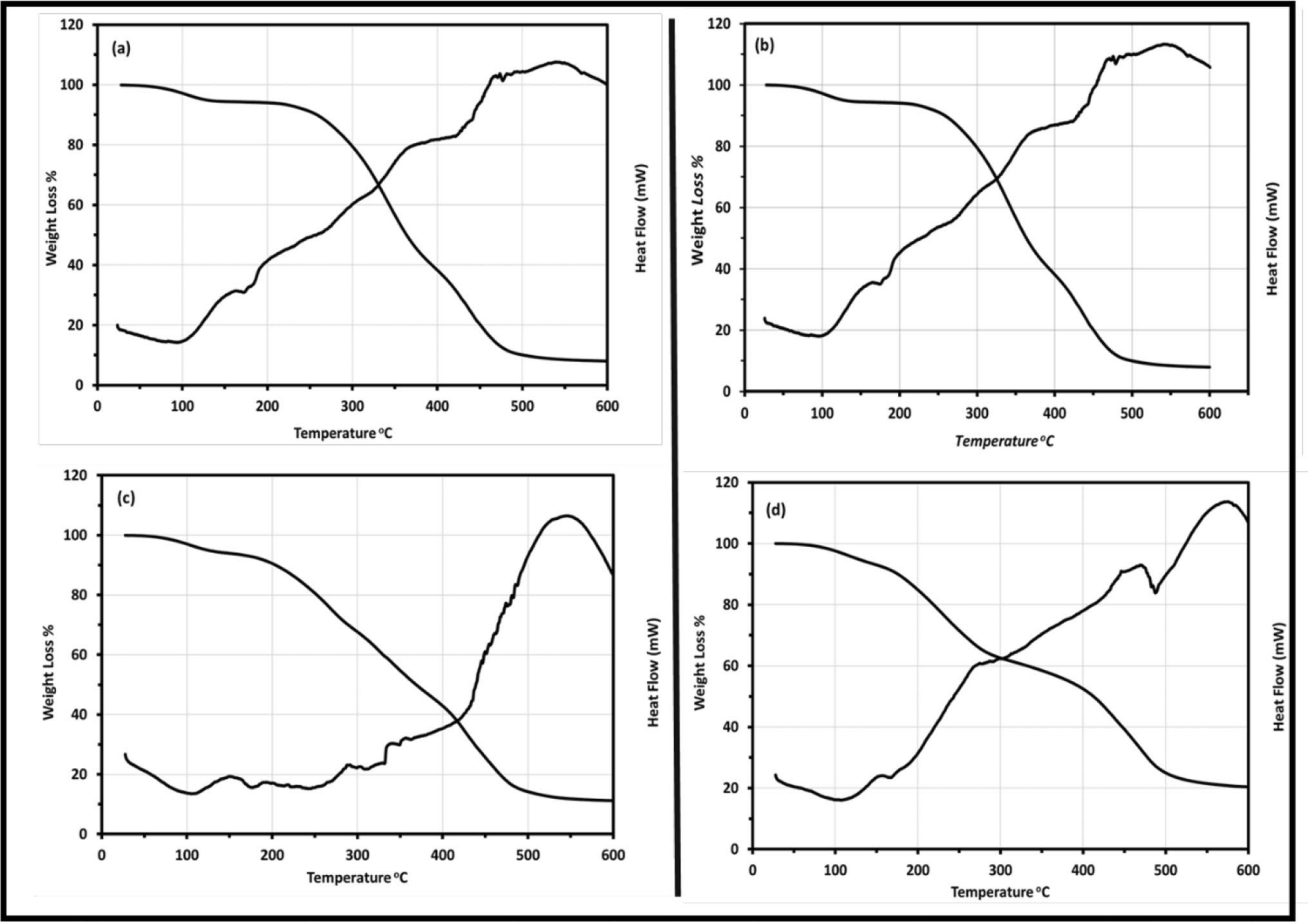
The TGA thermograms weight loss and DSC pattern versus temperature for the (PVA_1-x_PANi_x_) prepared films (where **a** x = 0, **b** x = 10, **c** x = 20, and **d** x = 30 wt. %)

Significant changes in the surface appeared with the PANi introduction in the PVA matrix. PANi nanofibers formation observed in the SEM images showed a mixture of nanofibers with slightly white porous spongy morphology patches as seen in Fig. 5C, D [53, 54]. This transition of different shapes with change in PANi concentration is visible from scanned images. The change is due to the change of micellar shape from hexagonal to spherical and again back to hexagonal with an increase of aniline of the PANi concentration [21]. Besides, the incorporation or the crosslinking of PANi into the PVA was shown in the film’s images because of the influence of the aniline concentration in the films developing some cracks or unevenness. Attentively, there are no separate domains for conducting and insulating components visible, which conduct interlayered association of the blend’s components and a unique chemical homogeneity of the prepared film which confirmed the formation of a homogenous interpenetrating network of polymer blends and the uniform dispersion [52–54].

The elemental composition of the prepared films was analyzed by energy-dispersive X-ray (EDX) analysis based on the SEM images of 1000 X magnification. The EDX spectrum disclosed only the existence of carbon, oxygen, nitrogen, and chlorine, and no impurities could be observed in the spectrum of samples, as shown in Table 2.

**Table 2:** EDX analysis elements weight for the prepared films

Elements	0 wt.%	10 wt.%	20 wt.%	30 wt.%
	Mass (%)	Mass (%)	Mass (%)	Mass (%)
C	42.31	39.24	35.18	31.38
N	–	8.61	12.39	10.53
O	57.69	50.41	50.34	56.51
Cl	–	1.74	2.09	1.59

### Thermal analysis

Thermogravimetric analysis (TGA) is considered the most important method to predict and study the thermal stability of polymer blends up to elevated temperatures. Figure 5 shows the thermogravimetric (TGA) and the Differential scanning calorimetry (DSC) patterns for the prepared films by heating the samples from room temperature up to 600 °C with a heating rate of 10 °C/min under a nitrogen atmosphere. Three thermal regions characterized the weight loss pattern. From the TGA curves, the weight loss in the first region occurs up to 100 °C, the second region observed between 100 and 250 °C, and the major weight loss happened in the third region between 250 °C and 450 °C. However, the final decomposition was implemented mostly after the 450 °C.

In the first region, the weight loss corresponds to loss of the adhered moisture, released free acids like HCL, unreacted monomer, and the dehydration of the polymer blends due to the presence of the hydroxyl group at every alternate carbon atom [21, 47, 54]. The second region resulted from the partial decomposition of the polymer blends with partial weight losses of 9% for the pure PVA film and 11% for the doped 10 wt.% PANi film, 19% for the doped 20 wt.% PANi film, and 29% for the dopped 30 wt.% PANi films, at 250 °C. The weight loss may be attributed to the loss of free hydroxyl functional groups due decomposition of the PVA long chains to short ones, the degradation of the short polymer, or the oligomer degradation produced during the polymerization process and the breaking of free amine functional groups associated to the decomposition of the PANi polymeric chain, which continues to gradually decrease along with the dehydroxylation of the copolymer up to 420 °C [47, 55, 56].

The maximum and major weight loss occurred at the third region (250–450) °C where the second stage of the thermal decomposition is clear. This weight loss is mainly due to the degradation of Coulomb attraction between the sulphonic acid and PANi backbone, decomposition, and cleavage of (C–C) bonds that occurred to form carbonate in the polymer structure and the degradation of the PVA molecules, resulting in the formation of water, acetaldehyde, acetone, ethanol, unsaturated aldehydes, ketones, and aromatic products. Besides, a gradual loss of hydroxyl and amine functional groups can be related. Furthermore, the molecular interaction (e.g., hydrogen bonding) amongst the acid, PVA, and PANi is no longer effective at this stage; therefore, a major weight loss takes place [21, 56, 57].

The activation energy of the decomposition for the prepared films depends on the residual mass of the composites and can be calculated by using the first-order integral equation of Coates and Redfern [58]:

4 $${\mathbf{Log}}\left[ {\varvec{ }\frac{{ - {\mathbf{Log}}\left( {1 - {\varvec{\alpha}}} \right)}}{{{\varvec{T}}^{2} }}} \right] = {\mathbf{Log}}\frac{{\varvec{R}}}{{\Delta {\varvec{E}}_{{\varvec{a}}} }}\left[ {1 - \frac{{2{\mathbf{RT}}}}{{{\varvec{E}}_{{\varvec{a}}} }}} \right] - \varvec{ }\frac{{{\varvec{E}}_{{\varvec{a}}} }}{{2.304{\mathbf{RT}}}}$$

where T is the absolute temperature, E_a_ is the activation energy in KJ/mol, R is the universal gas constant (8.3136 kJ/mol), and α is the fractional weight loss, which can be obtained as follows:

5 $${\varvec{\alpha}} = \varvec{ }\frac{{{\varvec{w}}_{{\varvec{i}}} - {\varvec{w}}_{{\varvec{t}}} }}{{{\varvec{w}}_{{\varvec{i}}} - {\varvec{w}}_{{\varvec{f}}} }}$$

where $w_{i}$ is the initial weight, $w_{t}$ is the weight at a given temperature and $w_{f}$ is the final weight of the sample. By fitting Eq. (5), we obtained the activation energies from the slopes of the straight lines. The thermal activation energies in the second and third thermal regions are listed in Table 3. The activation energies in the second thermal region increased from 25.96 to 51.16 kJ/Mol for x = 0 and x = 30 wt. %, respectively indicating the increasing of the thermal stability of the blends with increasing the PANi content. However, it decreases with the PANi introduction from 81.9689 to 51.89 kJ/Mol in the third thermal region which may be attributed to the amorphous structure of the PANi which the XRD and FTIR confirmed.

**Table 3:** Thermal parameters activation energy E_a_ (KJ/Mol), the residual weight at 250–450 °C, and the phase transition temperature for all prepared samples

PANi wt.%	Thermal Activation Energy E_a_ (*kJ* Mol^−1^)		Weight Loss (%)		The Phase Transition Temperature	
	Region II	Region III	250 °C	450 °C	T_g_	T_m_
0	25.96	81.70	9.5%	86.0%	85 °C	190 °C
10	35.14	81.39	9.0%	79.0%	100 °C	172 °C
20	42.32	63.86	20.0%	25.5%	115 °C	180 °C
30	51.16	51.89	29.0%	61.0%	125 °C	170 °C

Differential scanning calorimetry (DSC) is the most used method to determine thermal transition temperatures, such as glass transitions *T*_*g*_, melting point *T*_*m*_, crosslinking, and decomposition. From the DSC figures, the moisture release and impurities elimination endotherm appear around 70–85 °C, and the degradation and crosslinking of the polymer occurred above 250 °C forming polyene structures and volatile products for the pure PVA film in Fig. 5a [54]. Above the 450 °C, PVA decomposes into carbons and hydrocarbons [21]. For the PANi dopped films in Fig. 5b–d, a weak endotherm around 90–110 °C corresponded to the HCL releasing and moisture, followed by a broad endotherm around 400–450 °C corresponds to the morphological changes and the complete decomposition of the PANi dopped films [59, 60]. The phase transition temperatures are listed in Table 3, the *T*_*g*_ of the PVA is 85 °C, which gradually increases with increasing the PANi content in the blend. Generally, the shifting trend of glass transition temperature *T*_*g*_ can be considered as the index of miscibility of polymer blends, however, the melting temperature decreasing trend is due to the PANi amorphous nature which affected the blend behaviour [54, 60].

### DC electrical conductivity

**Fig. 6:**
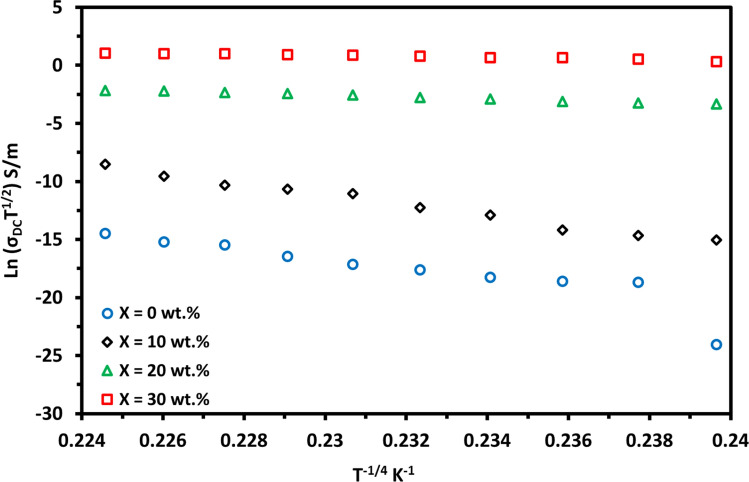
The temperature dependence of DC conductivity for the prepared blend films

Figure 6 illustrates the temperature dependence of out-of-plane DC conductivity, σ_DC_, carried out for (PVA_1-x_/PANI_x_) films in the temperature range 303–393 K. It is clear that the σ_DC_ increases steadily with temperature showing semiconductor behaviour as in the case of semiconductor materials. Temperature increase would lead to a decrease in the resistance of the polymer blend films, and thus the DC electrical conductivity was increased which can be attributed to the aggravated thermal motion of the molecules which increased the carrier concentration with the elevated temperature [30]. At room temperature, the σ_DC_ increases with increasing the PANi content in the polymer blend from 2.16 × 10^−12^ S.m^−1^ to 0.08 S.m^−1^ at for pure PVA film and 30 wt.% PANi polymer blend film, respectively as mentioned in Table 4. This increment is a result of mainly two shares; (1) the increase of the charge carrier concentration, besides, (2) the formation mechanism, where the good dispersion of the PANi on the films increased and facilitates the π − π interaction between the PVA and the PANi backbone, leads to a higher conjugated system. It is reported that interchain and intrachain hopping plays a major effect on the charge carrier transport inside the polymers as well as the carrier mobility is mainly dependent on the engineering, conformation, and arrangement of polymer chains. The prepared polymer blend is fabricated of mostly insulator polymer PVA and percentage weights of the conductive polymer PANi which has a preferably oriented and amorphous structure. Therefore, the highly oriented polymer chains can reduce barrier hopping, resulting in the easier movement of carriers and consequently increased electrical conductivity [40].

**Table 4:** Electrical Thermoelectrical parameters for (PVA_1-x_PANi_x_) films (where x = 0, 10, 20, and 30 wt. %) at room temperature

PANi Wt.%	At Room Temperature								
	$\sigma_{DC}$*(S.m*^*−1*^*)*	*T*_*0*_* (K)*	*N(E*_*f*_*) (eV*^*−1*^*.cm*^*−1*^*)*	R (A^0^)	W (eV)	S (µVK^−1^)	P.F (µW.m^−1^.K^2^)	ZT	K (W.m^−1^ K^−1^)
0 wt.%	2.08 × 10^−12^	9.94 × 10^07^	6.92 × 10^19^	53.98	0.16	–	–	–	0.1304
10 wt.%	1.67 × 10^−08^	6.03 × 10^07^	1.14 × 10^20^	47.64	0.14	340.68	1.933 × 10^−9^	2.33 × 10^−12^	0.26
20 wt.%	0.0021	3.41 × 10^07^	2.02 × 10^20^	41.33	0.12	74.12	1.11 × 10^−5^	1.18 × 10^−8^	0.286
30 wt.%	0.08	1.89 × 10^07^	3.65 × 10^20^	35.63	0.10	− 18.93395	2.85xx10^−5^	2.75 × 10^−8^	0.362

The PANi contains metallic regions considered “Conductive Islands” with multi-amorphous regions, with good conducting properties. These metallic regions ‘‘conductive islands’’ are well dispersed in the insulated areas of the PVA matrix. A few conducting paths are formed because of the low density of conductive islands. However, the PANi introduction into the blend, the more metallic regions formed in the blend due to the preferable alignment of polymer chains which makes the carrier transmission more convenient and increasing the electrical conductivity accordingly [40, 41].

The conduction mechanism was investigated using several models applied to the DC electrical conductivity experimental data at the temperature range (303–393 °C). The best fitting for the experimental data was shown by Greave’s model. According to the Greaves model, the conductivity is attributed to the hopping of charge carriers in three-dimensional between localized states at the Fermi level. It can be expressed by [34, 42]:

6 $${\varvec{\sigma}}_{{{\mathbf{DC}}}} \left( {\varvec{T}} \right) = \varvec{ \sigma }_{0} {\varvec{T}}^{ - 1/2} {\mathbf{exp}}\left( { - {\varvec{T}}/{\varvec{T}}_{0} } \right)^{1/4}$$

where $\sigma_{0}$ is a constant independent of temperature and *T*_*o*_ is the Mott characteristics temperature which has the formula:

7 $${\varvec{T}}_{0} = \varvec{ }16/\left( {{\mathbf{KN}}\left( {{\varvec{E}}_{{\varvec{f }}} } \right){\varvec{L}}^{3} } \right)$$

where *L* is the localization length and N(E_f_) is the density of states and is estimated by assuming an *L* value of 3 ^o^A for the blend. The estimated values of the *N(E*_*f*_*)* are listed in Table 4, the N(E_f_) increases with the introduction of the PANi content in the blend. This may contribute to the charge carrier’s concentration increasing with PANi content increases which enhances the density of states as well as the enhancement in the amorphous of the blend with increasing the concentration of the PANi as confirmed by the XRD diffraction, FTIR, and TGA.

The mean hopping distance R_hopp_ between two adjacent sites through a barrier height W_hopp_ is calculated by the following equations [42]:

8 $${\varvec{R}}_{{{\mathbf{hopp}}}} = (3/8) {\varvec{L}}\left( {{\varvec{T}}_{0} /{\varvec{T}}} \right)^{1/4}$$

9 $${\varvec{W}}_{{{\mathbf{hopp}}}} = (1/4)\varvec{ }{\mathbf{KT}}\left( {{\varvec{T}}_{0} /{\varvec{T}}} \right)^{1/4} { }$$

The *R*_*hopp*_ and *W*_*hopp*_ for *(*PVA_1-x_PANi_x_*)* blend films decreased with increasing PANi content showing the opposite trend of the density of states because of the crystallinity decrement, the charge carriers increment, and mobility as listed in Table 4.

### Thermoelectric parameters

**Fig. 7:**
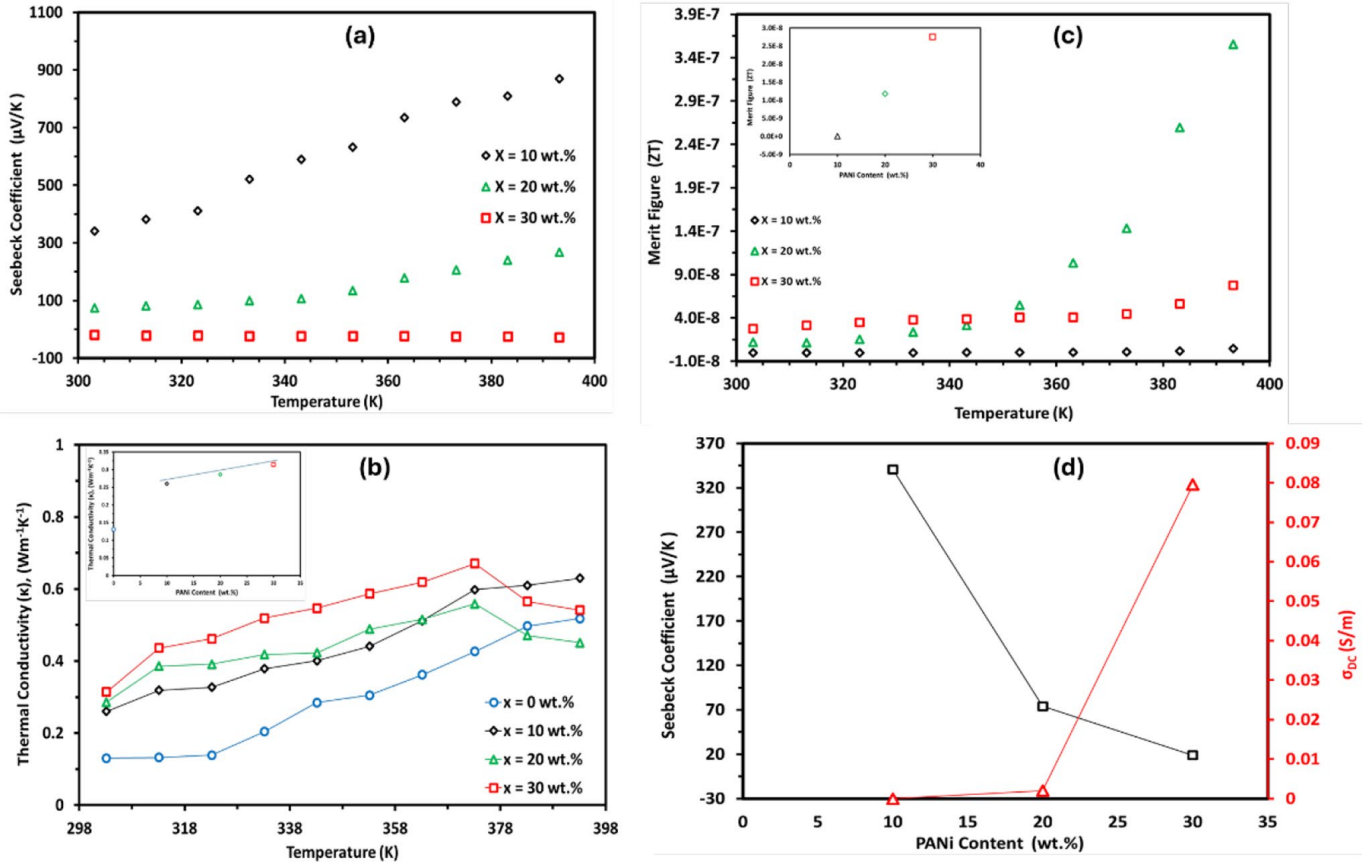
The temperature dependence of; **a** Seebeck Coeffienet (S), **b** thermal conductivity (K), **c** Merit figure; and **d** the Seebeck coefficient-DC electrical conductivity PANi concentration dependence of the prepared blend films

In Fig. 7a, The Seebeck Coefficient (S), temperature dependence, was figured for (PVA_1-x_PANix) where X = 10, 20, and 30 wt.% PANi. The films were sandwiched between a 4-prob structure with a temperature gradient. The temperature differences were controlled on the platform temperature from zero up to 10 with 10 steps of temperature gradient fields, and the temperature differences stabilized at each step for some time for measurements. According to the output voltages (emf) which were probing directly at the edges of samples, the Seebeck voltages between the ends of the samples were obtained. The Seebeck coefficient of the samples was calculated by Eq. (2).

As depicted in Fig. 7a, there is a significant decrease in the Seebeck coefficient with increasing the PANi content from 381 µV/K to − 18.9 µV/K for the 10 wt. % and 30 wt.% films, respectively. The Seebeck coefficient decrease is attributed to the increase in the charge carrier concentration with the PANi introduction [61, 62]. The positive sign of the low PANi-loaded films denotes the P-type semiconductor nature of the PANi [62]. However, the negative sign of the 30 wt.% films has been attributed to the electronic structure of the polymers, the increasing of the PANi metallic regions’ “conductive islands” in the prepared films which strongly affect the Seebeck coefficient and electrical conductivity as well as the PANi-type transition from *n*- to *p*-type [37, 63]. The Seebeck composition dependency behavior is opposite to the electrical conductivity as observed in Fig. 7d. Zhang B. et al., have reported the behavior that electrical conductivity and Seebeck coefficient cannot increase simultaneously due to narrow energy transport level (*E*_*T*_) and Fermi level (*E*_*F*_) and the polymer structure of the PANi [64, 65]. The Seebeck coefficient temperature dependency shows a clear increase with increasing temperature. The enhancement of the Seebeck coefficient can be explained through the mechanism of energy filtering of charge carriers [37, 66].

Figure 7b shows the temperature dependence of thermal conductivity for all the prepared films in the temperature range (303–393 K). The thermal conductivity of materials, κ, consists of two contributions: the lattice contribution from phonon transport, κ_L_, and the electrical contribution from electron/hole transport, κ_E_, as the following formula [66];

10 $$\kappa = \kappa_{L} + \kappa_{E}$$

where,

11 $$\varvec{\kappa ~}_{\varvec{E}} = \varvec{~L}\sigma _{{{\mathbf{DC}}}} \varvec{T}$$

where (L) is Lorenz number (2.44 × 10^−8^ W/S.K^2^), T is the temperature, and σ_DC_ is the electrical conductivity. In the analyses of thermoelectric properties on non-degenerate semiconductors, Eq. (10) has commonly been adopted to derive the lattice thermal conductivity from the measured thermal and electrical conductivity.

The thermal conductivity increased with the temperature. At room temperature, blend films’ thermal conductivity is slightly increased by increasing the PANi content of the polymers’ blend. Generally, the carrier mobility of conductive polymers is much lower than that of metals due to the structure of conductive polymers. The charge carrier contribution to the thermal conductivity is generally small for conductive polymers, while the phonon contribution is dominant [67, 68]. Numerous interfaces may act as phonons’ effective scattering centers where the phonons are highly scattered. Thermal transport in films is impeded, while a hopping transport mechanism can maintain the electrical conductivity. This is consistent with the low thermal conductivity and high electrical conductivity obtained experimentally for (PVA_1-x_PANi_x_) polymer blend films. The output power of the thermoelectrical cell is calculated by the power factor (PF) formula [62]:

12 $${\varvec{P}}.\varvec{F } = {\varvec{S}}^{2} {\varvec{\sigma}}_{{{\mathbf{DC}}}}$$

*where S* is the Seebeck coefficient, and σ_DC_ is the DC electrical conductivity. Figure 7c shows the Merit Figure temperature dependence for the investigated blend films. Further, the temperature increases, the Merit Fiure (ZT) increasing reaching 3.86xx10^−7^ at 393 K for the 20 wt.% PANi film as a result of increasing the electrical conductivity, and the low thermal conductivity of the prepared films.

Table 4 shows the thermoelectrical parameters for the prepared films at room temperature. With the PANi introduction, the power factor (P.F) increases from 1.933 × 10^−9^ (µW.m^−1^.K^2^) to 2.85xx10^−5^ (µW.m^−1^.K^2^) for the 10 wt.% and 30 wt.% PANI films, respectively. The ZT increased from 2.33 × 10^−12^ for the 110 wt.% PANi film to 2.75xx10^−8^ for the 30 wt.% PANi film. The Seebeck coefficient data is higher than that reported by other systems such as Ayat Abd-Elsalam et al. [41] and Reza Amirabad et al. [42], where S is about 141 µV/K and 11.4 μV/K at 338 K, respectively.

## Conclusion

The conjugated blend films of PVA/PANi have been synthesized by casting technique and chemical polymerization of the aniline. This research addresses the challenges associated with balancing the conflicted features of the high electrical conductivity characteristic of metals with the low thermal conductivity typical of insulators, thereby advancing the development of effective thermoelectric materials. The introduction of the PANi enhanced the structure and the thermal properties of the blended films by performing strong electrostatic interactions between the PVA and PANi. The structured molecular arrangement of the PVA/PANi polymer chain, combined with the amorphous nature of the blend, enhances electrical conductivity while regulating thermal conductivity. The DC Electrical conductivity increased sharply reaching 0.08 S.m^−1^ at room temperature and 1.44 S/m at 393 K for the 30 wt.% PANi film. The increase in the PANi content correlates with heightened carrier concentration and mobility, leading to an upward trend in electrical conductivity, a modest increase in thermal conductivity, and a decrement in the Seebeck coefficient. The 30 wt.% PANi film shows the optimal values for DC electrical conductivity, power factor, thermal conductivity, and the Merit Figure. These findings suggest that the lightweight and cost-effective PVA/PANi film holds considerable promise for thermoelectric applications, particularly in low-temperature environments.

## Data Availability

All data supporting this study and its findings are available within the article. Any data deemed relevant are available from the corresponding author upon request.
